# Intramedullary Spinal Tuberculoma: An Uncommon Presentation of a Common Disorder

**DOI:** 10.7759/cureus.28761

**Published:** 2022-09-04

**Authors:** Samra Iftikhar, Nadeem Ijaz, Sidrah Iftikhar, Shandana Khan

**Affiliations:** 1 Department of Radiology, Khyber Teaching Hopsital, Peshawar, PAK; 2 Department of Surgery, Khyber Teaching Hospital, Peshawar, PAK; 3 Department of Medicine, Hayatabad Medical Complex Peshawar, Peshawar, PAK; 4 Department of Radiology, Northwest General Hospital and Research Centre, Peshawar, PAK

**Keywords:** central nervous system tuberculosis, magnetic resonance imaging, hepatitis b infection, miliary tuberulosis, intramedullary spinal cord tuberculoma

## Abstract

Intramedullary tuberculoma (IMT) is rare and usually indistinguishable from spinal cord tumors. Thus, the diagnosis of an IMT is challenging. Our case deals with an unusual presentation of a 55-year-old Asian man who had presented with lower limb weakness which was found to be caused by the dissemination of tuberculosis (TB) resulting in an IMT, a rare complication of tuberculosis. The patient also had a concurrent incidental hepatitis B infection. The treatment of IMT is anti-tuberculous medication. This case highlights the significance of the prompt diagnosis of an IMT, urgent intervention particularly in developing areas of the world where tuberculosis is still endemic, an increased probability of patients having an IMT, and their diagnoses being missed.

## Introduction

Tuberculosis is the ninth leading cause of death worldwide [1]. Tuberculosis presenting as an intramedullary lesion within the spinal cord is rare as it occurs in approximately 1-2/100,000 patients with tuberculosis [2]. Intramedullary tuberculomas constitute about 0.2% to 0.5% of all central nervous system (CNS) tuberculomas [3]. About 170 cases of isolated IMT have been reported to date with the thoracic spinal cord being the most common site of involvement ­­­[4]. Intramedullary involvement by TB usually presents as transverse myelitis, radiculomyelitis, intraspinal granulomas, or thrombosis of the anterior spinal artery. Clinically, a spinal IMT mimics a spinal cord tumor. If diagnosed in a timely manner and managed promptly, it has a good prognosis [5]. This case describes a patient presenting with miliary TB and an IMT which is a rare complication of the primary disease.

## Case presentation

A 55-year-old male with no previous co-morbidities presented to the medical outpatient department with low-grade fever, cough, and two episodes of shortness of breath for the last 8-10 days. He also has a history of bilateral lower limb weakness for almost three months which was initially noted in the left leg and then gradually involved the right leg. He had documented low-grade fever on/off, associated with night sweats and occasionally dry cough with subjective weight loss. The relevant examination findings are depicted as follows (Table 1).

**Table 1:** Initial examination findings. mmHg: millimeters of mercury, °F: degrees Fahrenheit, F: Fahrenheit, S1: First heart sound, S2: Second heart sound

Examination	Examination Findings
Glasgow Coma Scale Score	15/15
Temperature (°F)	100
Central Nervous System	Oriented to person, place and time
Heart	S1+S2+0 no murmur
Heart Rate (beats/minute)	96
Blood Pressure (mmHg)	130/90
Chest	Bilateral basal crepitations, more on right side than the left
Respiratory Rate (breaths/min)	22
Saturation (SpO2 %)	90
Abdomen	Non-distended, soft and non-tender
Lower Extremity Motor	Decreased strength in lower limbs (3/5), and loss of tone in the bilateral lower extremities

The initial lab investigation showed neutrophilic leukocytosis, raised C reactive protein, raised erythrocyte sedimentation rate, and hypoalbuminemia. Virology report revealed a positive Hepatitis B surface antigen which was confirmed with a positive Hepatitis B PCR. The laboratory investigations are summarized in Table 2.

**Table 2:** Laboratory findings. mcL: microliter, %: percentage, g/dL: grams/deciliter, mg/L: milligrams/liter; g/dL: grams/deciliter, mm: millimeters, mg/dL: milligram/deciliter, HBeAg: Hepatitis B e-antigen, IU/mL: international units/milliliter, HBV: Hepatitis B virus, PCR: Polymerase chain reaction

Laboratory Investigations	Reference Limits	Results
Total Leukocytes (10^3^/mcL)	4.0-11.0	15.1
Neutrophils (%)	40-75	84
Lymphocytes (%)	20-45	10
Monocytes (%)	06-10	08
Eosinophile (%)	01-06	02
Hemoglobin (g/dL)	Male: 13-18	13.1
Platelets (10^3^/mcl of blood)	150-450	366
ESR (mm/1^st^ hour)	Male: 0-15	62
C-Reactive Protein (mg/L)	<5.0	9.61
Total Serum Protein (g/dL)	6.6-8.7	5.9
Serum Albumin (g/dL)	3.5-5.0	2.60
Bilirubin (mg/dL)	0.1-0.25	0.54
Prothrombin Time (seconds)	10	11.8
Activated Partial Thromboplastin Time (seconds)	28	28
HBeAg IU/ml	Cut-off index for non-reactive < 1.0 IU/ml	Reactive (13.79)
HBV PCR (Quantitative)	>500 IU/ml	99883
Liver Function Tests	Normal	
Renal Function Tests	Normal	

**Figure 1:**
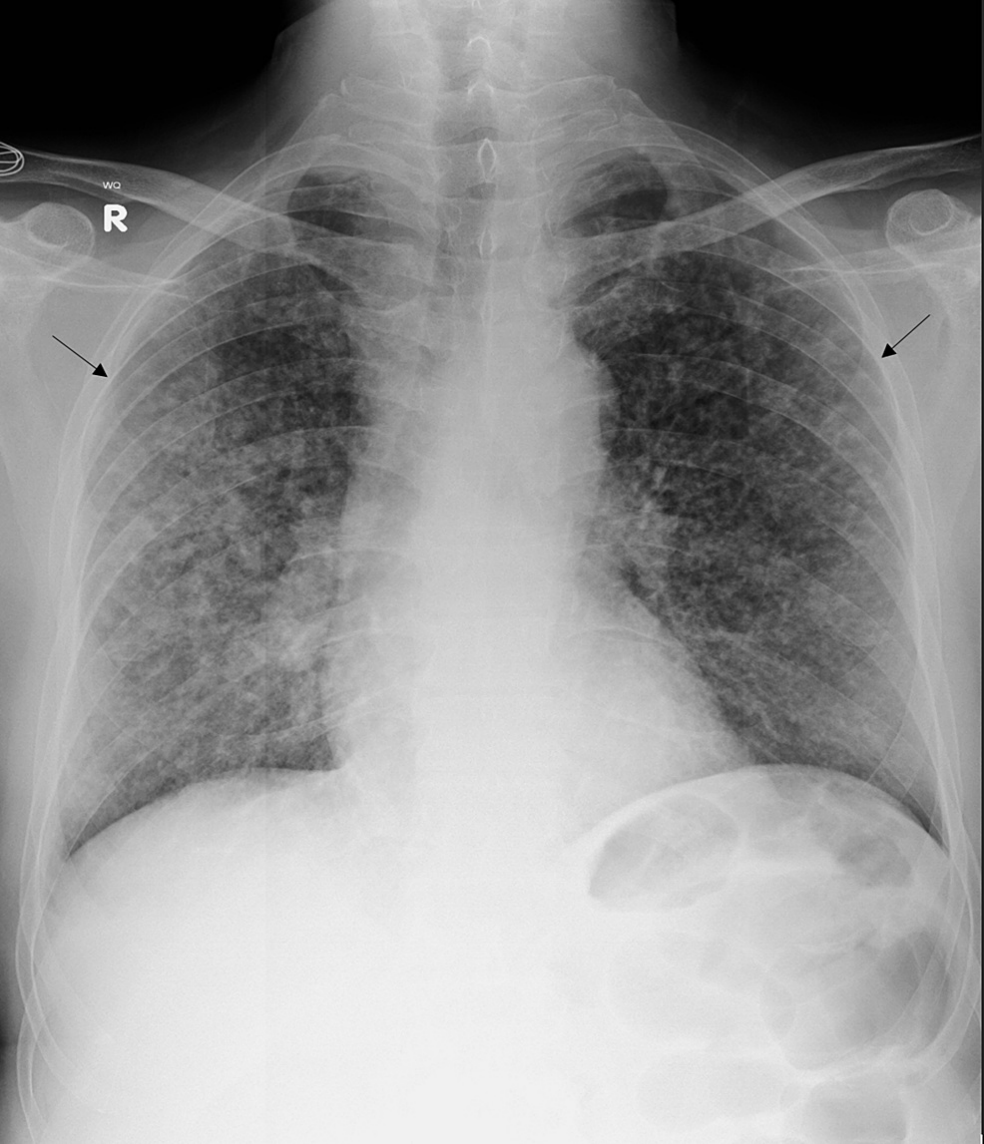
The patient's chest x-ray (PA view) showed multiple small reticulonodular nodules are seen throughout all lung lobes bilaterally. PA: Posterioanterior.

He was referred to the Radiology Department for a chest x-ray, computed tomography (CT) chest with contrast, and magnetic resonance imaging (MRI) spine to evaluate the probable cause of his symptoms. The chest X-ray showed numerous small reticulonodular soft tissue densities bilaterally with an enlarged right hilar lymph node indicative of miliary tuberculosis (Figure 1).

**Figure 2:**
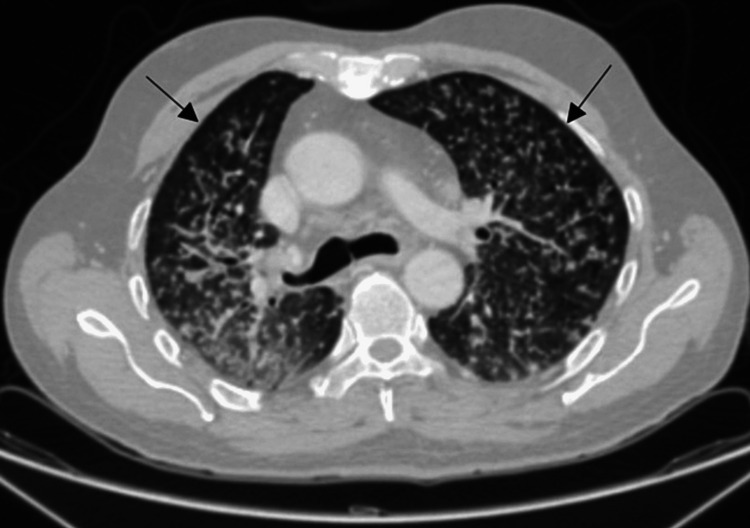
The chest CT with contrast, axial view (lung window), depicted multiple centri-lobular tree in bud nodulations. CT: Computed Tomography

CT chest with contrast showed multiple randomly distributed nodules throughout the lung parenchyma with some being centrilobular (tree-in-bud) and others about the pleura. Additionally, enlarged lymph nodes were noted along the right hilum and azygo-esophageal recess. Subcentimeter lymph nodes were also seen along the aortic arch and aortopulmonary window with a bilateral pleural reaction, supporting the diagnosis of miliary tuberculosis (Figure 2).

**Figure 3:**
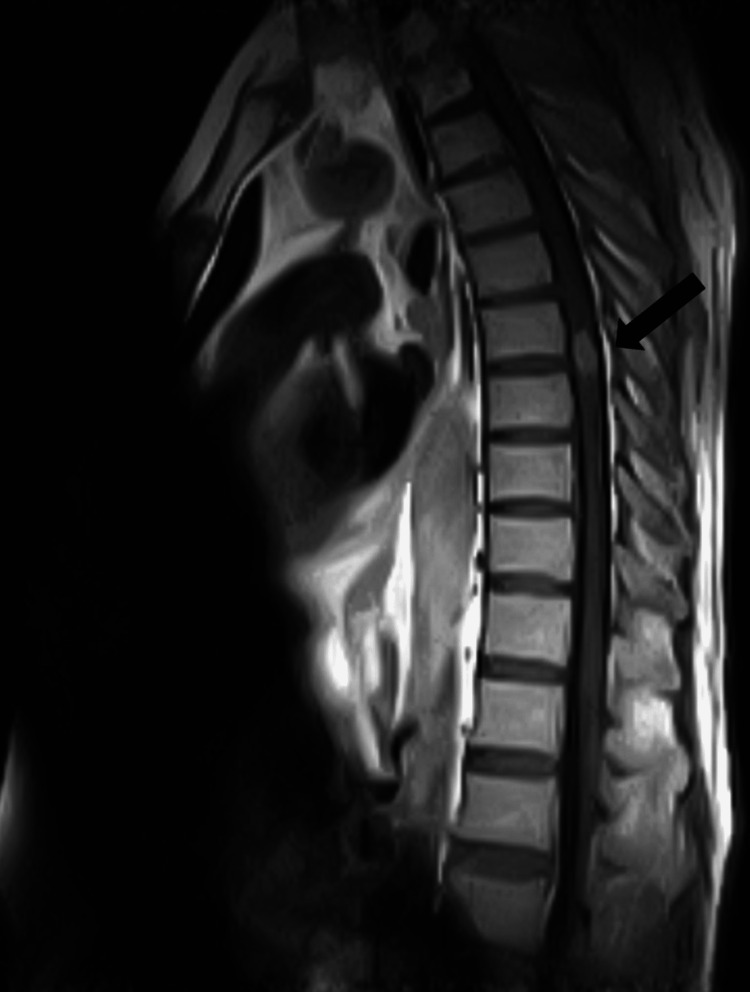
The sagittal T1-weighted post contrast thoracic MRI shown above illustrated a single enhancing lesion at T7/T8. MRI: Magnetic Resonance Imaging, T: thoracic

**Figure 4:**
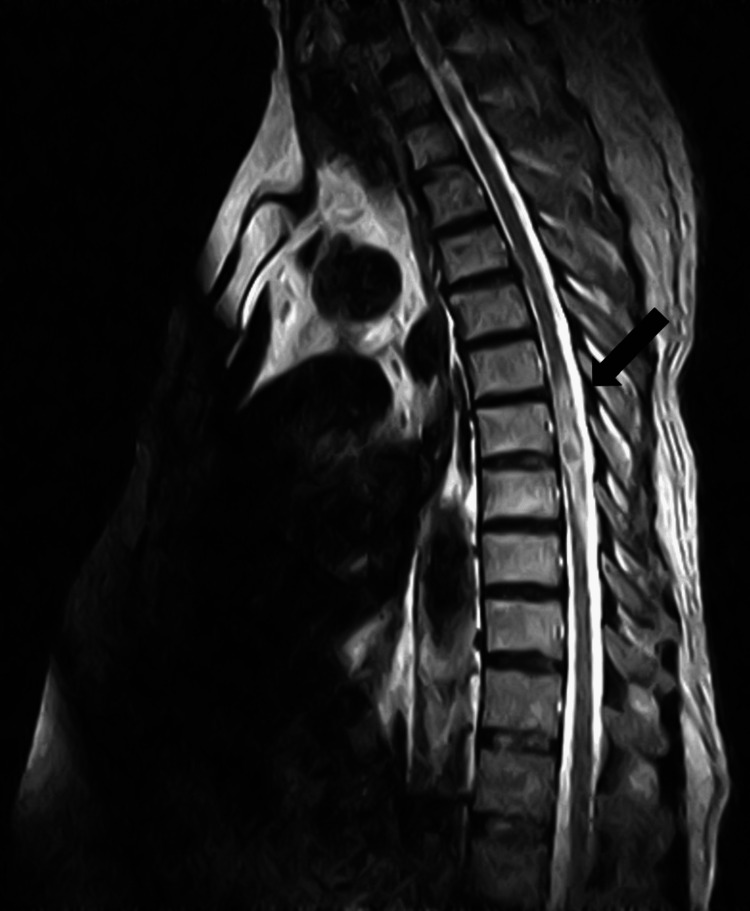
The sagittal T2-weighted thoracic MRI seen above showed a central low signal intensity lesion with a peripheral high intensity rim and surrounding edema in the cord at the T7/T8.

**Figure 5:**
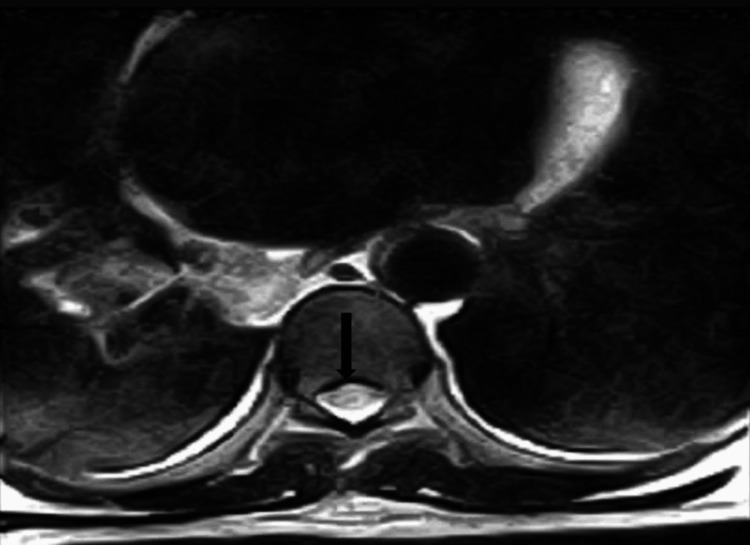
The axial T2-weighted thoracic MRI documented ring enhancement with a central low signal intensity intramedullary lesion.

**Figure 6:**
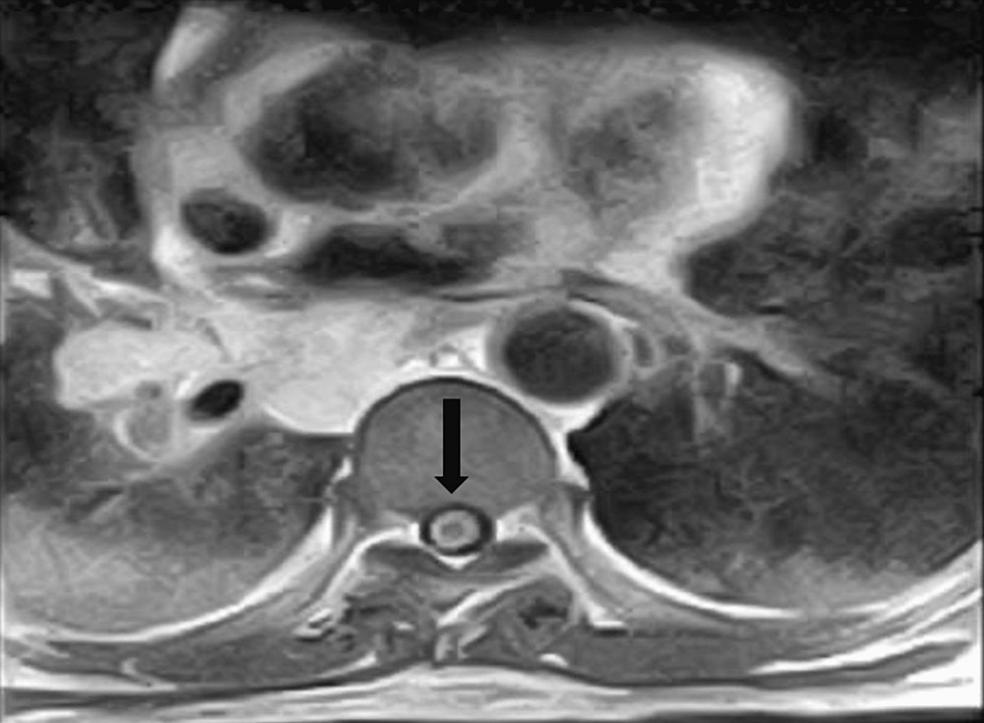
The axial T2-weighted thoracic MRI confirmed an intramedullary hypointense lesion with peripheral enhancement.

The thoracolumbar spine MRI was requested to evaluate for his bilateral lower extremity weakness and revealed a well-defined small intramedullary space-occupying lesion located at T7/T8 level measuring 12 x 9 x 8 mm. This lesion was low on T2-weighted imaging with the mild expansion of the cord and extensive surrounding cord edema. Post-contrast sequences revealed peripheral contrast enhancement of the lesion (Figures 3-6).

The patient was placed on the standard anti-tuberculous regimen of rifampicin, isoniazid, pyrazinamide, and ethambutol (RIPE) along with supplemental pyridoxine. For better CNS penetration, oral steroids were commenced in a tapering dose, and Entecavir was also part of the treatment regimen since the patient also had a hepatitis B infection. He was discharged from the hospital and a one-month follow-up was advised. The patient was lost to follow-up, however.

## Discussion

The CNS involvement in TB in the form of a tuberculoma is rare as it occurs in approximately 10% of the cases and concurrent involvement of the spine having a tuberculoma is extremely rare [6]. The first case of intramedullary spinal TB was noted in 1828 by Abercombie et al. a constitute of two in 1,000 CNS TB cases [7]. Nussbaum et al. reported that only 7% of IMT were seen in a series of 29 cases reported over a 20-year period [8]. Of countries with a high TB burden, Pakistan has the 11th highest number of cases. In spite of this high incidence and burden, there is a paucity of information on IMT in the adult population in Pakistan [1]. There is not sufficient data available to prove whether IMT is associated with pulmonary tuberculosis or not.

Spinal IMT is mainly the consequence of the hematogenous spread of a primary infection or cerebrospinal fluid seeding. However, in a few cases, it can result from the local spread of pulmonary TB. Cases of isolated IMT are rare, and current reports suggest that silent dissemination of primary TB could potentially be responsible [9]. It is essential to confirm the presence of primary TB which was evident in our patient based on findings of miliary TB on chest X-ray. Since he was having untreated pulmonary TB, he was at risk of developing extrapulmonary TB as a result of hematogenous spread. Although chest x-rays and high-resolution CT scans are normally used to diagnose primary disease, MRI is the preferred modality for investigating a tuberculoma anywhere in the body.

It is important to identify the characteristic features of intramedullary spinal TB on MRI as it has other similarly presenting differential diagnoses like transverse myelitis or gliomas. During the initial phase of tuberculoma formation, there is extensive edema with a severe infection and the signal characteristics are a hypointensity on both T1-weighed and T2-weighed sequences. As time progresses and tuberculoma maturation occurs, there is cord expansion and hypointensity of the central tuberculoma present on T2-weighted sequence with a ring enhancement surrounding the hypointense area on post-contrast images. Our case presented with the characteristic findings of tuberculoma on a thoracolumbar MRI, i.e., a central low signal intensity lesion with surrounding high-intensity signal edema on T2-weighted sequence and ring-enhancement on post-contrast images. A ring-enhancing lesion with a hypointense center on a T2-weighted image, also known as the “target sign” on MRI points toward the diagnosis of IMT [10]. Although, confirmation of the diagnosis, however, needs histopathological evidence of caseating granulomas along with a mycobacterium on culture results and acid-fast bacilli documented on Ziehl-Neelson stain [11].

Anti-tuberculous therapy is the mainstay therapy for IMT. Surgical resection might be an option in patients with an increasing size of a tuberculoma which is causing significant neurologic deficit [12]. The prognosis of spinal TB has now improved with early diagnosis and rapid intervention, however, spinal tuberculomas are a rare presentation of tuberculosis even in areas where the disease is endemic. A high degree of clinical suspicion is required in patients presenting with signs and symptoms suggestive of radiculopathy. Medical treatment is generally effective. This case is quite unusual as the patient was hepatitis B positive whereas previous studies suggest that it is more common for patients having human immunodeficiency virus (HIV) or immunocompromised individuals to develop tuberculous complications. Whether there is an increased propensity to develop extra-pulmonary TB in the setting of hepatitis B infection needs further research. The limitations of our study were that the patient was lost to follow-up and there was a lack of availability of histopathological evidence to confirm the diagnosis of IMT. The classic MRI findings along with widespread pulmonary disease make IMT the obvious diagnosis.

## Conclusions

This case is unusual because it emphasizes the importance of imaging with correct interpretation and having a low threshold for MRI of the spine in patients presenting with weakness who have been diagnosed with disseminated TB. Diagnosing IMT can be challenging as they appear similar to other lesions, present with nonspecific symptoms, and occur in the general population rarely. The association of these tuberculomas with hepatitis B or C infections needs further research.
